# Wharton’s jelly and osteoarthritis of the knee

**DOI:** 10.1093/bmb/ldad030

**Published:** 2023-12-07

**Authors:** Adarsh Aratikatla, Nicola Maffulli, Manu Gupta, Ishana A Potti, Anish G Potty, Ashim Gupta

**Affiliations:** The Royal College of Surgeons in Ireland, Dublin D02 YN77, Ireland; Department of Musculoskeletal Disorders, School of Medicine and Surgery, University of Salerno, Fisciano 84084, Italy; Department of Trauma and Orthopaedics, Ospedale Sant’ Andrea, Sapienza University of Rome, Rome, Italy; Barts and the London School of Medicine and Dentistry, Centre for Sports and Exercise Medicine, Queen Mary University of London, London E1 4DG, UK; School of Pharmacy and Bioengineering, Keele University School of Medicine, Stoke on Trent ST5 5BG, UK; Polar Aesthetics Dental & Cosmetic Centre, Noida, Uttar Pradesh 201301, India; South Texas Orthopaedic Research Institute (STORI Inc.), Laredo, TX 78045, USA; South Texas Orthopaedic Research Institute (STORI Inc.), Laredo, TX 78045, USA; South Texas Orthopaedic Research Institute (STORI Inc.), Laredo, TX 78045, USA; Future Biologics, Lawrenceville, GA 30043, USA; BioIntegrate, Lawrenceville, GA 30043, USA; Regenerative Orthopaedics, Noida 201301, India

**Keywords:** umbilical cord, Wharton’s jelly, regenerative medicine, perinatal tissue, osteoarthritis, knee osteoarthritis

## Abstract

**Introduction:**

The existing treatment modalities for knee osteoarthritis (OA) do not actually address the pathology. Biological therapies, including those using material derived from perinatal tissues, represent a ground-breaking approach to alleviating the symptoms of OA of the knee.

**Source of data:**

Current scientific literature published in PubMed (MEDLINE), Embase and Scopus databases. Trials registered in various clinical trial databases.

**Areas of agreement:**

Perinatal tissues including Wharton’s jelly (WJ) and associated mesenchymal stem cells (MSCs) can be used for the management of knee OA.

**Areas of controversy:**

The efficacy of WJ and associated MSCs in the management of knee OA is still controversial.

**Growing points:**

The use of WJ and associated MSCs in the management of knee OA is safe and appears to be effective.

**Areas timely for developing research:**

The present published evidence suggests that WJ tissue and associated MSCs offer an encouraging alternative for the management of knee OA. The published *in vitro*, preclinical and clinical investigations demonstrate the therapeutic potential of WJ and promote further research in this field to provide symptomatic relief to patients suffering from OA, aiming also to regenerate the osteoarthritic hyaline cartilage, with disease-modifying effects. Future adequately powered randomized controlled trials should be undertaken to establish whether WJ is helpful in the management of OA of the knee.

## Introduction

Knee osteoarthritis (OA) is the most common joint disorder in the USA.^1^ Its pathogenesis can be attributed to either primary causes (i.e. non-traumatic/idiopathic) or secondary degeneration of the joint from malalignment or trauma.^2^ Knee OA accounts for over 80% of all OA cases worldwide, affecting over 30 million individuals in the USA alone.^3^^,^^4^ Many current forms of treatment tend to provide symptomatic relief, not focusing on the underlying pathology of OA.^5^ Recommended lifestyle changes include increased exercise, dietary counselling, bracing and physiotherapy.^6^^,^^7^ Further examples of non-surgical interventions include, but are not limited to, non-steroidal anti-inflammatory drugs (NSAIDs), corticosteroid injections, nutritional supplements and viscosupplementation.^8^ Although both NSAIDs and corticosteroid injections provide short-term relief, chronic use may lead to complications and further degeneration of the joint tissue in the case of corticosteroids.^9^^,^^10^ Glucosamine sulphate and chondroitin sulphate have shown some beneficial therapeutic effects, but more clinical studies and research are necessary to determine whether they have a significant positive impact on patients with knee OA.^11^ Viscosupplementation (i.e. hyaluronate injections) provides temporary symptomatic relief, but the outcomes are inconsistent.^12^^,^^13^ The aforementioned treatments only provide transient symptomatic relief.

Autologous and allogenic biologic sources address some of the limitations caused by traditional modalities. Biologic therapies include platelet-rich plasma adipose tissue and bone marrow–derived stem cells.^14^ Perinatal tissues for orthobiologic use can be sourced from the amnion/chorion membrane, amniotic fluid, umbilical cord (UC) and umbilical cord–derived Wharton’s jelly (UC-WJ) along with the mesenchymal stromal/stem cells associated with these tissues.^15^

The human UC connects the mother’s placenta to the foetus. It can be divided into three parts: the amnion, blood vessels and Wharton’s jelly (WJ). WJ can be further subdivided into the subamnion, intermediate WJ and perivascular WJ.^16^ WJ is a muco-gelatinous connective tissue composed mostly of extracellular matrix rich in collagen fibres (i.e. Type I/II mostly), glycosaminoglycans, proteoglycans, myofibroblasts and diffuse plasma proteins.^16^^,^^17^ WJ supports and protects the umbilical vasculature by preventing torsion and compression.^18^ WJ provides a rich supply of mesenchymal stem/stromal cells (MSCs) and yields the highest concentration of MSCs per millilitre when compared to other allogenic tissue sources, making it an abundant source for biologic use.^19^ It is easily obtained, as WJ is usually collected without harming the donor and is often discarded after delivery.^20^ We systematically review the *in vitro*, preclinical and clinical outcomes of WJ tissue and the associated MSCs in the management of OA of the knee. The secondary objective is to document the ongoing clinical trials registered on various trial protocol repositories on WJ and its use to manage knee OA.

## Materials and methods

### Search criteria

**Fig. 1:**
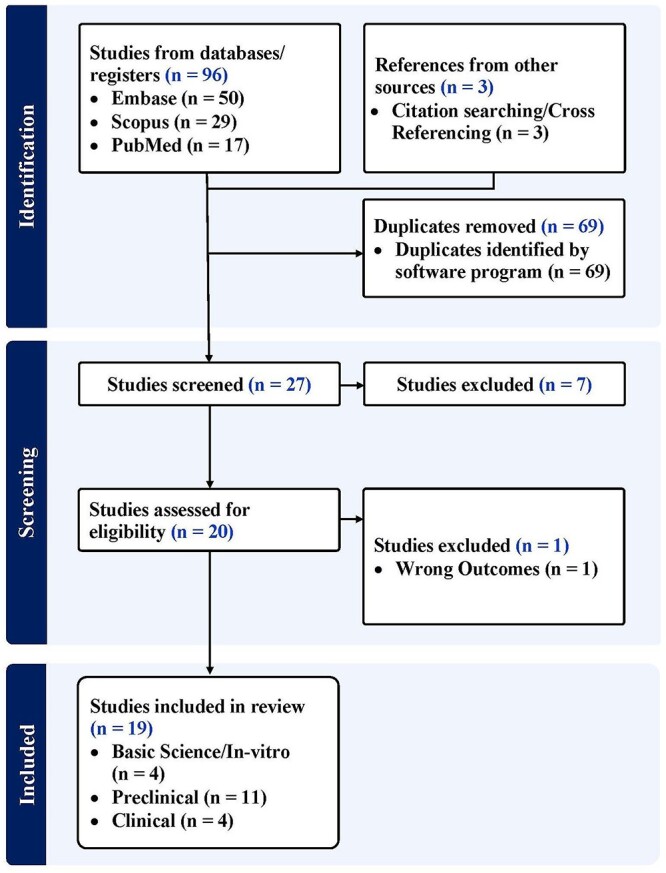
PRISMA flow diagram.

A systematic search was performed using PubMed (MEDLINE), Embase and Scopus databases for articles published in English before November 2023. The Preferred Reporting Items for Systematic Reviews and Meta-analyses (PRISMA) statement and guidelines were followed, using the following search terms: (‘Wharton’s jelly’ OR ‘Whartons jelly’ or ‘Wharton jelly’) AND (‘knee OA’ OR ‘knee osteoarthritis’) using PubMed, Embase and Scopus databases. Studies were eligible if they involved acute and chronic OA of the knee and animal or human knee OA models. Publications must have used tissue, or MSCs, derived from UD-WJ as an intervention. Placebos, non-injury models, acute injury models, non-injury models and gold-standard treatments were compared. The study selection was performed by two independent reviewers using a dedicated reference management software. Figure 1 illustrates the systematic search performed.

In addition, we searched ClinicalTrials.gov, Chinese Clinical Trial Register (ChiCTR) and Clinical Trials Registry—India (CTRI) using the same search terms to identify registered trials on the use of WJ in the management of OA of the knee.

## Results

### *In vitro* studies

Sofia *et al*. determined the effect of WJ-MSCs on the expression of prostaglandin E2 (PGE2), which plays an essential role in the development of knee OA, in synoviocytes *in vitro*. There were six groups in this study: two control groups and four experimental groups. Groups I and II contained synoviocytes only and were incubated for 24 and 48 hours, respectively. Groups III and IV contained WJ-MSCs incubated for 24 and 48 hours, respectively. Groups V and VI were co-culture groups, containing both osteoarthritic synoviocytes and WJ-MSCs, incubated for 24 and 48 hours. WJ-MSCs were administered onto synovial tissue from patients with knee OA post-total knee replacement (TKR). The WJ-MSC and synoviocyte co-culture group demonstrated the most significant reduction in Prostaglandin E2 (PGE2) expression at 24 and 48 hours, compared to the control groups and WJ-MSCs only.^21^^,^^22^

Furthermore, another investigation was conducted at the same institution to determine the influence of WJ-MSCs on matrix metalloproteinase-13 (MMP-13) and RELA (v-rel avian reticuloendotheliosis viral oncogene homologue A) gene expression, using the same study design. Similar to what was reported for PGE2, WJ-MSCs exhibited a statistically significant decrease in the gene expression of MMP-13 and RELA compared to the controls. For MMP-13, control groups I and II demonstrated gene expressions of 1.00 and 1.00 ng/μl, respectively, while co-culture groups V and VI showed gene expressions of 0.13 and 0.04 ng/μl, respectively. For RELA, control groups I and II demonstrated gene expressions of 1.00 and 1.00 ng/μl, respectively, while co-culture groups V and VI demonstrated gene expressions of 0.16 and 0.16 ng/μl, respectively. WJ-MSCs can therefore potentially decrease gene expression of MMP-13 and RELA, both of which are implicated in the progression of OA. WJ-MSCs therapy may help delay OA progression in the knee.^23^

Kusuma *et al*. investigated the effect of conditioned medium (CM) from insulin-like growth factor 1 (IGF1)-induced human WJ-MSCs (hWJMSC) to manage knee OA. WJ cells were isolated and then treated with IGF1, which promotes the chondrogenic differentiation of stem cells. The CM was incubated and subsequently collected. To determine the therapeutic potential of the CM, the researchers used human chondrocytes to institute an *in vitro* model of OA and added CM to the chondrocyte model. Six groups of cells were involved in this study: chondrocytes only, chondrocytes with interleukin-1-beta (IL1-beta), chondrocytes with IL1-beta and WJ-MSC-CM at 15% or 30% concentrations and chondrocytes with IL1-beta and IGF1-treated WJ-MSC-CM at 15% or 30% concentrations. Various analytic studies evaluated its effects on chondrocyte proliferation, extracellular matrix (ECM) synthesis and cellular viability. IGF1-hWJMSCs-CM enhanced the proliferation and viability of chondrocytes when compared to the control groups. CM also increased the production of aggrecan and type II collagen, both necessary to maintain the structure and function of cartilage. The study showed that the biologic significantly increased the gene expression of COL2, an essential protein in the cartilage matrix, suggesting its potential for repairing damaged chondrocytes in OA. MMP gene expression was also reduced, showing that IGF1-hWJMSCs-CM may exert a protective effect against cartilage degeneration. Cell signalling pathways controlling cartilaginous homeostasis and chondrogenesis (i.e. Akt, ERK, etc.) were activated by CM. In conclusion, IGF1-hWJMSCs may be used to treat OA, as they promote their sustenance and inhibit the expression of degradative cytokines. Further studies are warranted to determine whether these stem cells are effective in an *in vivo* model of OA.^24^

Muthuchamy *et al*. aimed to determine whether human WJ-MSCs could be used as a scaffold in combination with autologous chondrocytes to promote cartilage repair in patients with OA. The scaffold was composed of WJ, after the MSCs had been extracted, and was then seeded with autologous chondrocytes obtained following a minimally invasive procedure. Once cultured in a viable medium, chondrocyte proliferation and differentiation characteristics were assessed. The UC-derived WJ scaffold supported cartilaginous attachment, when used with autologous chondrocytes, demonstrating the potential of WJ to be used to regenerate articular cartilage.^25^

### Preclinical studies

Cheng *et al*. inoculated rat knees with an intraarticular (IA) injection of monosodium iodoacetate to induce early OA. In addition to the WJ-MSC and control group, another experimental group was included, where extracorporeal shockwave therapy (ESWT) was used. The group treated with WJ-MSCs received an IA knee injection of 1 × 10^6^ WJ-MSCs into the knee joint, with the control group injected with saline. The rats were assessed at various intervals for joint function, response to pain, histological changes and expression of regenerative markers. WJ-MSCs improved the pain response and knee function in early OA. The rats given the biologic therapy showed increased ability to weight bear on the affected knee and reduced pain responses when compared to the control specimens. Focusing on specific markers suggestive of the therapeutic potential of WJ-MSCs, the researchers observed increased expression of type II collagen and growth factors, such as TGFβ-1 and IGF-1. TUNEL expression was also decreased, indicating a favourable effect on chondrocyte apoptosis.^26^^,^^27^

Endrinaldi *et al*. evaluated the effect of WJ-MSCs on ADAMTS-4 and iNOS levels in a rat OA model. ADAMTS-4 serves as an aggrecanase, an enzyme responsible for the degradation of aggrecan—an integral component crucial for the maintenance and support of chondrocyte viability.^28^ iNOS plays an inflammatory function in OA, as it produces nitric oxide.^29^ Researchers induced OA into a rat model by surgically destabilizing the medial meniscus (DMM). Specimens were divided into a control group, which received saline, and a WJ-MSC group (1 × 10^6^ cells), which was injected directly into the affected knees immediately after the destabilization surgery. At predefined time intervals, ADAMTS-4 and iNOS levels were assessed using biochemical and immunohistochemistry assays. ADAMTS-4 and iNOS levels demonstrated a statistically significant reduction, indicating that this biologic therapy can potentially regress the enzymatic degeneration that OA exerts on the knee joint. The anti-inflammatory potential of WJ-MSCs must be further studied to ascertain whether these stem cells have a beneficial effect on the progression of OA.^30^

Xing *et al*. determined whether IA WJ-MSCs therapy can slow the progression of knee OA in rats (*n =* 18). Anterior cruciate ligament (ACL) transection and medial meniscectomy were performed in rats, which were subsequently divided into three groups: one control, one injected with hyaluronic acid and one with WJ-MSC (1 × 10^6^ cells). Parameters assessed included behavioural therapy, micro-computer tomography (CT), macroscopic appearance, histological analysis and a modified Mankin score, which assesses the severity of cartilage degeneration. Macroscopic evaluation suggested that OA may be suppressed by this therapy, if administered during the early stages of OA. The histological state of the medial femoral condyles in all three groups was evaluated using the modified Mankin score. While the HA + MSC group still exhibited significant cartilaginous and proteoglycan loss, the combined therapy group demonstrated minimal indications of OA progression.^31^

Li *et al*. investigated the efficacy of a composite scaffold composed of WJ and chondroitin sulphate (CS), a key element found in the cartilage extracellular matrix (ECM), loaded with human UC-MSCs (hUC-MSCs), to help regenerate cartilage defects in rats’ knees. A medial parapatellar approach was used to induce a full-thickness articular cartilage defect via a corneal trephine in the rat knees. Histology was used to score the amount of regeneration, where 0 represented complete regeneration and 14 represented no regeneration.^32^ The rats were divided into three groups: WJ + hUC-MSCs + CS, WJ + hUC-MSCs and an untreated control group. The WJ + hUC-MSCs + CS group demonstrated both the highest amount of type II collagen formation and the lowest histological scores compared to the other groups. Immunofluorescence, histology and gene expression analysis showed that the composite scaffold with hUC-MSCs produced a statistically significant improvement compared to the other control groups. WJ + hUC-MSCs + CS exert a strong favourable chondrogenic response, it is unclear whether they will be effective in humans.^33^

Saulnier *et al*. studied the effects of xenogeneic neonatal UC-MSCs given IA 3 and 15 days after OA had been induced in the knees of 30 rabbits via a medial meniscal release (MMR). Evaluations were performed on Days 15 and 56, to determine whether any significant differences were present between the early and mid OA time points. The MSCs (3.5 × 10^6^ cells), derived from the WJ of an UC, were successfully isolated and cultured, exhibiting fibroblastic morphology. They expressed commonly seen MSC markers (CD90, CD29, CD44, CD105 and MHC1), while lacking CD45 and MHC2 expression. *In vitro* differentiation tests demonstrated their potential to differentiate into osteogenic, adipogenic and chondrogenic lineages, as represented by calcium accumulation, lipid droplet formation and glycosaminoglycan (GAG) synthesis, respectively. Gross morphological assessment of the knee joints confirmed the presence of osteoarthritic lesions on the tibial plateau, with early synovial fibrosis, osteophyte formation and cartilage damage. These findings were consistent with early OA lesions at Day 15 and mild OA at Day 56. The transected meniscus did not heal in any of the knees, but the early treatment group exhibited lower osteophytic and cartilage damage scores, particularly on the medial tibial condyle, compared to the control and delayed treatment groups. A few rabbits in both treatment groups did experience mild synovial inflammation, as histology showed the presence of lymphoplasmacytic cellular infiltrates at Day 15 and Day 56. Regarding gene expression, there was significant up-regulation of IL1-β and TNF-α at Day 15. IL10. MMP-1, -3 and -13 expression were not affected after administration of UC-MSCs. At Day 56, the number of inflammatory mediators was decreased in both treatment groups compared to the control. This study demonstrates the efficacy of early IA-injected, xenogeneic UC-MSCs in preventing the progression of OA in rabbits following MMR, but these are preclinical observations, and it is unclear whether these findings apply to the human clinical setting.^34^

Zhang *et al*. evaluated the effect of canine MSCs on dogs with knee OA confirmed with imaging. The knee OA was surgically induced into the canines, and the WJ-derived MSCs (WJ-MSCs; 1 × 10^6^ cells) were isolated using collagenase I, confirmed via immunofluorescence and then tested for osteogenic differentiation. On Days 1 and 3 after treatment, a suspension of allogeneic MSCs and an equal amount of saline were injected into the joint of the treated and control groups, respectively. The structure of the dogs’ knee joint was assessed via magnetic resonance imaging (MRI), ultrasound (US) and fluoroscopy 3, 7, 14 and 28 days after the procedure. IL-6, IL-7 and TNF-α in the dogs’ blood were measured. After 35 days, electron microscopy was used to compare the recovery in cartilage and patellar surface between the treatment and untreated groups. The isolated cells were confirmed as UC-derived WJ-MSCs based on surface markers (i.e. CD44+ and CD34−) and their osteoblastic differentiability. At imaging, the treated group had improved, showing a decrease in high signal on MRI, disappearance of echo-free space on US and thickening of the hypoechoic region. Furthermore, plain radiographs showed improvement in patellar defects and increased density at the ventral patella and trochlear crest. In contrast, the control group showed increasing high signal and echo-free space, along with discontinuous hypoechoic regions and no improvement in the patella defects. Inflammatory marker analysis demonstrated statistically significantly lower levels of IL-6, IL-7 and TNF-α in the treated group, when compared to the control group at 7–14 days after treatment. The thickness of the newly generated cartilage was significantly different between the treated group and the control group. Despite the positive effects of WJ on knee OA in dogs, further studies are warranted to confirm this in humans.^35^

Zhang *et al*. determined the effect of UC-derived WJ-MSCs (1 × 10^6^ cells) on cartilaginous repair following microfracture in a caprine model, when used in conjunction with an acellular cartilage ECM (ACECM). After human UCs were donated by consenting mothers, they were processed, cultured and harvested at ≈80% confluence. To better assess the chondrogenic and osteogenic potential of the hWJMSC, the stem cells were cultured in a chondrogenic and osteogenic medium, respectively, for 21 days. The chondrogenic pellets were then sectioned, fixed and stained with type II collagen and Alcian blue immunofluorescence to evaluate PG synthesis, using specific antibodies and fluorescent microscopy, while the osteogenic pellets went through the same process, but were stained with Alizarin red dye.^36^ Later in the experiment, MSCs were seeded onto an ACECM-oriented scaffold, which was constructed using ACECM suspension, obtained from articular cartilage. The resulting human WJ-MSCs/ACECM-oriented scaffolds were implanted into a femoral condyle defect of goats. The stem cells demonstrated an increased proliferative capacity and exhibited a standard fibroblast-like morphology with spindle cells, indicating promising cellular viability and a typical mesenchymal phenotype. Immunophenotyping confirmed significant expression of MSC surface markers (i.e. CD44, CD73, CD90 and CD105) at a high percentage (≥95%), while haematopoietic cell markers (i.e. CD34, CD45 and HLA-DR) showed minimal expression (≤2%). After 21 days of cultivation in the chondrogenic medium, there was increased GAG and glycoprotein deposition in the pellets, while increased collagen II expression was observed in the induction group compared to the non-induction group, indicating promising chondrogenic differentiation potential of the stem cells. Additional analysis demonstrated the presence of calcium nodules and some adipogenic differentiation. H&E staining of knee synovial fluid showed that the inflammatory reaction at 14 days was reduced compared to that at 7 days, but a statistically significant difference was not achieved.^36^ These stem cells demonstrated excellent chondrogenic differentiation potential and low immunogenicity, making them a hopeful candidate for cartilaginous regeneration. The use of an ACECM-oriented scaffold further promoted oriented cell growth and chondrogenic differentiation, without the need for an inducer. Compared to the microfracture technique, WJ-MSCs combined with the scaffold demonstrated superior regeneration outcomes, including the quantity and quality of new cartilage and the preservation of subchondral bone. This technique showed effectiveness and safety in a large animal model, suggesting its potential for biological cartilage repair in humans. Further research is still necessary to evaluate the immune response to xenogeneic stem cell transplantation and ensure the safety of WJ-MSCs in clinical applications.^36^

Dormer *et al*. studied the effect of UC-MSCs (20 × 10^6^ cells/ml) derived from the WJ of New Zealand White rabbits on osteochondral defects in rabbit knees. This study examined four treatment groups: sham surgeries without implants, blank scaffolds, gradient scaffolds with encapsulated growth factors and gradient scaffolds pre-cultured with rabbit UC-MSCs before implantation. Stem cells were harvested from rabbit UCs, incubated in collagenase, diluted, centrifuged and then plated. Once thawed, they were resuspended at a concentration of 20 × 10^6^ and placed on the scaffold, allowing for cellular attachment for 12 hours prior to surgical implantation. A defect in the medial femoral condyle was produced in 10 rabbits using a medial parapatellar incision. The experimental groups received 15 μl of the biologic solution. Plugs were press-fitted into the defects. Sham defects were also produced, whereby the defect in the articular cartilage of the medial femoral condyle was left unfilled. Rabbits received buprenorphine and were allowed unrestricted movement post-operatively. Histological analysis at 6 and 12 weeks revealed differences in cartilage regeneration in the experimental groups, with the sham group exhibiting complete filling of the defect and the sham group showing minimal cartilage regeneration. The gradient-only group showed better defect filling and neo-cartilage depth compared to the control group, while the gradient group with pre-cultured rUCMSCs did not show superior articular cartilage formation. At 12 weeks, all groups exhibited increased cartilage regeneration, with the sham, gradient-only and gradient + rUC-MSC groups performing better than the blank group. The presence of bioactive signals appeared essential to modulate osteoblastic activity by week 12, as demonstrated by the inferior regeneration in the blank group. The study suggested that bioactive signalling could be beneficial for osteochondral tissue regeneration. However, the healing process was delayed in all experimental groups compared to sham surgery, possibly from the higher molecular weight of the polymer used, which prolonged the mechanical integrity of the defect. Future studies should include larger sample sizes, animal models, defects and histological analyses to further explore the advantages of this stem cell in osteochondral regeneration.^37^

Endrinaldi *et al*. aimed to determine whether an optimal duration of WJ-MSC treatment could be attained when comparing the levels of MMP-1 and TGF-β in a rat model of OA (*n* = 30). Knee OA was produced by a single IA injection of iodoacetate. The rats were divided into the following five groups, and collection of the blood and knee joints occurred after the experiment: early OA, OA after 4 and 8 weeks, OA rat groups treated with WJ-MSCs for 4 and 8 weeks. The three groups that did not receive biologic therapy exhibited a statistically significant decline in articular cartilage thickness over the course of the study; furthermore, treatment with WJ-MSCs resulted in statistically significant increases in cartilage thickness. Although statistical significance was not achieved, the groups of rats not given the WJ-MSC therapy exhibited increasing levels of MMP-1, while a decreasing trend was observed after administering the therapy. A similar increasing trend was seen when measuring TGF-β levels in the rats not treated with the biologic, with statistical significance achieved when the WJ-MSCs therapy was used. WJ-MSCs may be an applicable treatment for knee OA, as histologically it showed promising results by the decreasing trends seen in TGF-β and MMP-1. Further preclinical and clinical studies should determine whether this is an effective biologic treatment to aid the progression of knee OA.^38^

A further study by Endrinaldi *et al*. aimed to determine the effect of WJ-MSCs on MMP-1 and IL-4 levels in knee OA in rats (*n =* 32). The rats were randomly and evenly divided into 2 groups, namely control and WJ-MSC. Histological, serum, and haematological analysis was subsequently conducted. Histopathology showed an increase in cartilage thickness and chondrocytic cell density in the WJ-MSC group compared to the control group. MMP-1 and IL-4 levels showed a statistically significant increase compared to the control. This study suggests that WJ-MSCs may have immunosuppressive or proinflammatory effects, as these markers increased after the biologic therapy.^39^

Kovalchuk *et al*. monitored the effects of transplanted human WJ-MSCs (1–1.5 × 10^6^ cells) in a rat OA model in which arthritis was induced through an IA injection of iodine–acetic acid. The rats displaying signs of OA were then selected for the study, while a control group of intact rats and sham-operated male rats received injections of saline. Positive CD markers included CD73, CD90 and CD105, while CD34 was negative. To evaluate the therapeutic potential of hWJ-MSCs for OA, these cells were introduced into the osteoarthritic knee joints of rats, and PCR analysis revealed their limited survival duration. However, the presence of a collagen scaffold prolonged the retention of the cells, suggesting that human WJ-MSCs hold promise as a viable cell source for human OA treatment. Further studies are necessary to conclude whether this is a practical biologic therapy to treat knee OA.^40^

### Clinical studies

Sadlik *et al*. evaluated the efficacy of MSCs derived from WJ of UC, when embedded into a collagenous scaffold implanted with dry arthroscopy. UC tissue cultures were processed, the fragments were washed, blood vessels were removed and WJ was sliced and cultured with a xeno-free medium. The cells were incubated, and their viability and characteristics were confirmed. The cells were then suspended in a mixture of cytoprotectant and human albumin, frozen and stored in liquid nitrogen. After thawing and washing out the cytoprotectant, the WJ-MSCs were collected, pelleted, resuspended and transferred into a syringe. Patients were positioned supine and anaesthetized, so a diagnostic arthroscopy could be performed to assess the areas of articular cartilage suitable to produce a contained lesion and undertake a subsequent repair. WJ-MSCs offer promising preclinical data and can be used in a single-stage technique of cartilage regeneration with careful monitoring and controlled rehabilitation. Safety assessments through MRI scans showed no adverse effects, but long-term follow-up is necessary. Despite the small patient cohort and lack of a control group, WJ-MSCs still offer a minimally invasive alternative to major surgery and contribute to the advancement of tissue engineering for articular cartilage repair, particularly in older patients with knee OA.^41^

Gupta *et al*. investigated knee OA patients using IA injection of UC-derived WJ formulation prepared and characterized following FDA and tissue bank standards in a 27-year-old male. The cord was processed aseptically without enzymes or cryoprotectants, with minimal manipulation, and underwent sterility testing before use. The patient had a history of ACL reconstruction and presented with pain during weight-bearing and physical activity. Baseline radiographs showed grade II OA with no progression, and IA injection of UC-derived WJ formulation resulted in a 50% reduction in pain at 3 months. The patient’s overall KOOS score increased by over 10%, with improvements in all subsets, and satisfaction levels increased from baseline to extremely satisfied. Short Form 36 Health Survey Questionnaire (SF-36) analysis showed a 25% improvement in overall health, with advances in physical function, role limitations from physical health, energy/fatigue, emotional wellbeing, pain and general health. The UC-derived WJ formulation is rich in bioactive factors and hyaluronic acid and has shown potential for regenerative therapy in knee OA. The formulation demonstrated safety and clinically significant improvements as assessed by NPRS, KOOS, 7-point Likert scale and SF-36.^42^^,^^43^

Gunay *et al*. assessed various outcomes post-IA injection of WJ-MSCs in 10 patients with knee OA who were administered WJ-MSCs after the failure of 6 months of conservative management. After the administration of the MSCs, patients were advised to restrict their activities for 7 days and apply a cold compress. All patients underwent clinical evaluations at each visit, using the visual analogue scale (VAS) to assess pain. Functional changes were evaluated using the WOMAC and Lequesne index, while changes in quality of life were assessed using the SF-36. Quantitative cartilage analysis was conducted based on the MRI Osteoarthritis Knee Score (MOAKS) cartilage segmentation, dividing the knee into 14 articular distinct regions. Cartilage thickness measurements were obtained from specific MRI images of different knee regions. TNF-α, IL-6 and -1β and various adipokines were analysed using enzymatically embedded immunoassays. IA injection of WJ-MSCs was found to be clinically beneficial, as indicated by decreased pain scores (VAS) and improved function (WOMAC), patient satisfaction (SF-36) and cartilage thickness in specific regions. Adipokine levels (i.e. adiponectin, leptin and resistin) showed significant changes post-injection, providing a cartilage-protective environment. Pro-inflammatory cytokine levels (IL-6 and -1β and TNF-α) also increased, reflecting the immunomodulatory effect of WJ-MSCs. The study was limited by its small sample size, short follow-up period, absence of a control group and lack of histopathological analysis, suggesting the need for further research with larger patient cohorts, histopathological analyses, extended observation periods and comparison to control groups.^44^

Samara *et al*. studied the effect of WJ-MSCs on knee OA to determine its efficacy. Patients of both genders (ages: 18–75 years old), with chronic knee joint pain for more than 6 months and confirmed Kellgren and Lawrence grade III/IV knee OA were included. Patients were excluded if they had knee effusions, significant malalignment, injury, deformity or previous IA treatment. Inclusion criteria also involved the absence of infection, haematological abnormalities, and atypical laboratory tests. Exclusion criteria encompassed various factors such as age, high BMI, whether the prospective female patient was of childbearing age, malignancy, using immunosuppressants or were scheduled for knee arthroplasty.^45^ Baseline and postoperative MRI scans used a semi-quantitative assessment form to assess various chondrocytic and osteocytic characteristics (i.e. cartilage damage, bone marrow lesions, osteophytes, subchondral sclerosis, joint effusion and synovitis). Each pathology was graded on an ordinal scale, and articular lesions were evaluated in 17 different regions. Osteophytes and bone marrow lesions were scored based on size, joint effusion based on width and synovitis based on the number of thickened villi. Subchondral sclerosis was recorded as either present or absent in the lateral, medial and patellofemoral knee compartments. A suprapatellar approach was used to inject WJ-MSCs under ultrasound guidance by an interventional radiologist IA. Improvement in cartilage damage, bone marrow lesions, synovitis, subchondral sclerosis and osteophyte development demonstrated statistically significant results between 6- and 12-month follow-up visits.^45^

### Ongoing clinical trials

As of November 10, 2023, seven clinical trials are registered on ClinicalTrials.gov to study the effects of WJ and associated MSCs for treatment of knee OA (search terms: Wharton’s jelly and knee osteoarthritis) and are summarized in Table 1. No trials were registered on the Chinese Clinical Trial Register (ChiCTR) or Clinical Trials Registry—India (CTRI) using the aforementioned search terms.

**Table 1:** Clinical trials registered on ClinicalTrials.gov till November 10, 2023, using WJ for the management of knee OA

Study identifier	Tissue/cell type	Study phase; estimated enrolment (*n*)	Primary outcome measure(s)	Recruitment status	Country
NCT04719793	UD-WJ	Early phase I; *n* = 12	1) Treatment-emergent adverse effects as assessed by comprehensive metabolic profile; creatinine levels; liver function test; complete blood count; C-reactive protein; erythrocyte sedimentation rate; T, B and NK cell lymphocyte subset; serum IgG, IgA, IgM and IgE levels (time frame: 1 week, 6 weeks, 3 months, 6 months, 12 months): To determine safety, i.e. adverse events associated with IA administration of CCM as assessed by comprehensive metabolic profile; creatinine levels; liver function test; complete blood count; C-reactive protein; erythrocyte sedimentation rate; T, B and NK cell lymphocyte subset; serum IgG, IgA, IgM and IgE levels.	Not yet recruiting	USA
NCT04711304	UD-WJ	Phase I/II; *n* = 168	1) Adverse or severe adverse events associated with IA administration of UD-WJ (time frame: immediately after injection, 24 hours, 48 hours, 1 week, 6 weeks, 3 months, 6 months, 1 year): To determine safety, i.e. adverse or severe adverse events associated with IA administration of UD-WJ injection. Any adverse or severe adverse events will be recorded in the associated case report forms. 2) Patient Satisfaction associated with IA administration of UD-WJ (time frame: 1 week, 6 weeks, 3 months, 6 months, 1 year after injection): To determine patient satisfaction via 7-point Likert scale. An increase in score indicates improvement. 3) Patient Satisfaction associated with IA administration of UD-WJ (time frame: change from baseline to 3 months after injection, change from baseline to 6 months after injection, change from baseline to 1 year after injection): To determine patient satisfaction via a 36-item short form survey (SF36).	Not yet recruiting	USA
NCT03866330	WJMSCs	Phase I/II; *n* = 100	*Only knee-specific outcomes listed. 1) Change in KOOS score. [time frame: baseline (T0), 3 months (T1), 6 months (T2), 9 months (T3), 12 months (T4), 24 months (T5)]. 2) Change in IKDC Questionnaire score. [time frame: baseline (T0), 3 months (T1), 6 months (T2), 9 months (T3), 12 months (T4), 24 months (T5)]. 3) Change in WOMAC score. [time frame: baseline (T0), 3 months (T1), 6 months (T2), 9 months (T3), 12 months (T4), 24 months (T5)]. 4) Change in Visual Analogue Scale (VAS) for pain in the target hip following completion of treatment cycles. [time frame: baseline (T0), 3 months (T1), 6 months (T2), 9 months (T3), 12 months (T4), 24 months (T5)].	Unknown	Poland
NCT02963727	WJMSCs	Phase I; *n* = 10	Evaluation of the safety and tolerability of the IA injection. Patients will be monitored for any adverse events resulting from the IA injection of WJMSC. (time frame: 6 months).	Unknown	Jordan
NCT04313894	WJMSCs	Phase II; *n* = 11	1) Changing in pain (time frame: before injection and 15 day—30 day—45 day—3 months—6 months—12 months after injection). 2) Changing in functional knee score using the Western Ontario and McMaster Universities Arthritis Index (WOMAC) (time frame: before injection and 15 days—30 days—45 days—3 months—6 months—12 months after injection). 3) Changing in quality of life. Short Form 36 (time frame: before injection and 15 days—30 days—45 days—3 months—6 months—12 months after injection). (time frame: before injection and 15 days—30 days—45 days—3 months—6 months—12 months after injection). 5) Chondral and subchondral changes. Magnetic resonance imaging T2 mapping (msn): (time frame: before injection and 15 days—30 days—45 days—3 months—6 months—12 months after injection). 6) Changes in leptin levels in synovial fluid (ELISA) (time frame: before injection and 15 days—30 day—45 days—3 months—6 months—12 months after injection). 7) Changing in adiponectin levels in synovial fluid (ELISA) (time frame: before injection and 15 days—30 days—45 days—3 months—6 months—12 months after injection). 8) Changes in tumour necrosing factor-α levels in synovial fluid (ELISA) (time frame: before injection and 15 days—30 days—45 days—3 months—6 months—12 months after injection). 9) Changing in resistin levels in synovial fluid (ELISA) (time frame: before injection and 15 days—30 days—45 days—3 months—6 months—12 months after injection). 10) Changing in interleukin-6 levels in synovial fluid (time frame: before injection and 15 days—30 days—45 days—3 months—6 months—12 months after injection). 11) Changing in interleukin-1β levels in synovial fluid (ELISA) (time frame: before injection and 15 days—30 days—45 days—3 months—6 months—12 months after injection).	Unknown	Turkey
NCT04520945	UC WJMSCs	Phase II; *n* = 100	1) VAS score. Change from baseline in VAS (time frame: 12 months). 2) WOMAC score. Change from baseline in Western Ontario and McMaster Universities Osteoarthritis Index Score (WOMAC) (time frame: 12 months). 3) IKDC score. Change from baseline in knee function change and improvement (IKDC) (time frame: 12 months). 4) KOOS score. Change from baseline in Knee Injury and Osteoarthritis Outcome Score (KOOS) (time frame: 12 months).	Unknown	Malaysia
NCT04863183	MSCs derived from WJ of UC	Phase I/II; *n* = 30	1) Decrease in joint pain. Pain will be measured through the VAS (time frame: 12 months). 2) Increased joint functionality. Joint functionality will be measured through the Western Ontario and McMaster Universities Osteoarthritis Index (WOMAC) (time frame: 12 months). 3) Improvement in the quality of life. Quality of life will be measured through the SF-36 (time frame: 12 months). 4) Imaging improvement of articular cartilage. The improvement of articular cartilage will be evaluated by magnetic resonance imaging (time frame: 12 months).	Unknown	Colombia

## Discussion

The present study evaluated WJ derived from the UC for the management of OA of the knee. *In vitro*, preclinical and clinical studies were qualitatively reviewed to assess their use in knee OA.

After investigating the impact of WJ-MSCs on the expression of PGE2, MMP-13 and RELA, which are strong indicators of OA progression, Sofia *et al*. suggested that a reduction in the expression of these pathogenetic markers in response to biologic treatment may have potential therapeutic benefits in attenuating the progression of knee OA.^21–23^ Kusuma *et al*. evaluated the potentially beneficial effects of WJ-MSCs when treated with IGF1, suggesting that this therapy may have a regenerative effect on chondrocytes, given the increased levels of SOX9, which, in turn, increases COL2 (i.e. a vital cartilage matrix protein) expression. IGF1 may have played a more prominent role in the restoration of cartilage cells compared to WJ-MSCs alone. The study also examined additional inflammatory markers, namely, TNF-alpha, IL10 and MMP-3. Notably, the application of IGF1-WJMSC-CM resulted in a substantial decrease of these marker levels by 15%, indicating the potential of this therapeutic approach in effectively mitigating inflammation associated with OA.

To gain more confidence in the therapeutic potential of WJ-MSCs, an additional study could include a group consisting solely of WJ-MSCs, without growth factors. This would provide more conclusive evidence regarding the independent efficacy of WJ-MSCs.^24^ Muthuchamy *et al*. determined that a decellularized human UC-WJ scaffold effectively supports a microenvironment necessary for the attachment of viable chondrocytes.^25^ Overall, only a few *in vitro* studies examined the specific effects of WJ-MSCs on knee OA, and future research should evaluate the immunomodulatory impact of these stem cells on knee OA. Overall, the basic science studies published so far have shown promising results, but further investigations should be conducted looking at additional metrics, such as inflammatory, degenerative, cell death markers to state more conclusively that this biologic therapy can produce beneficial results in patients with knee OA.

The preclinical studies identified describe the effects of WJ-MSCs on knee OA, usually induced by surgery on various animal models. Cheng *et al*. determined that a combination therapy utilizing ESWT and WJ-MSCs was beneficial in treating knee OA. Animal models of experimentally induced OA showed promising results, considering that one of the experiment groups containing only WJ-MSCs had a positive effect on MMP-13, SOX9 and RUNX-2 expression. WJ-MSCs therapy also showed better results compared to only shockwave therapy, as trabecular thickness and bone volume were increased with the use of WJ-MSCs. Interestingly, WJ-MSCs significantly decreased the amount of type II collagen, when compared to the shockwave and combined therapy group.^26^ Endrinaldi *et al*. conducted three further studies assessing the effect of WJ-MSCs on specific inflammatory marker levels in rats with knee OA. In one experiment, the levels of ADAMTS-4 and iNOS, which are degradative and inflammatory molecules, respectively, were examined. Treatment with WJ-MSCs resulted in a statistically significant reduction in the levels of both.^30^ Another trial showed that WJ-MSCs had a net positive effect on MMP-1 and TGF-beta levels.^38^ A further investigation by the same group confirmed that the MMP-1 and IL4 markers were elevated after the introduction of WJ-MSCs compared to the control group.^39^ The conflicting outcomes may result from a need for standardized testing methods, indicating a need for additional meticulously designed studies to definitively establish the suitability of WJ-MSCs as a viable biologic for the management of knee OA. Xing *et al*. showed that WJ-MSCs may slow osteoarthritic progression, as confirmed after molecular, histopathological and macroscopic analyses.^31^ Li *et al*. concluded that a composite scaffold may promote cartilaginous regeneration when used in conjunction with WJ-MSCs, as it provides a strong foundation for structural integrity and adherence to the surrounding tissues. The stem cells showed superior integration to the surrounding cartilage tissue, and the chondrocytic arrangement was similar to that of normal tissue.^33^ Saulnier *et al*. demonstrate the impact of a single IA injection of xenogeneic UC-MSCs in preventing signs of OA in rabbits after a medial meniscal release. The timing of the IA injection plays a crucial role, with earlier administration showing superior outcomes. Delayed injection was associated with increased inflammation and further progression of OA. MSCs can therefore exert their bioactive potential across species and modify genes associated with matrix turnover and inflammation in synoviocytes. A healthy microenvironment may be eventually restored if endogenous cells are recruited, as they may play a role in local immunomodulation. These results should be interpreted cautiously, as cross-species interactions may result in a strong detrimental immune response.^34^ Bei-ying Zhang *et al*. showed that canine UC-MSCs promote cartilage repair in patella OA, reducing the localized inflammatory response and slowing the effect of the injury to the adjacent tissues. The treatment group in this study exhibited better recovery in the skin fascia, muscle and cartilage compared to the control group.^35^ Yu Zhang *et al*. determined that the WJ-MSC combined with the acellular cartilage ECM showed better regenerative capacity compared to the microfracture group. The functional and structural integrity of subchondral bone was better maintained when compared to microfractures. Additionally, this synergistic effect resulted in a better arrangement of articular cartilage chondrocytes, closer to their physiological appearance. Overall, WJ-MSCs showed promising regenerative effects, seen through their low immunogenicity and capability for chondrogenic differentiation.^36^ Dormer *et al*. not only studied the regenerative effect of the WJ-MSCs but also assessed the regenerative effect of the gradient, sham and blank implants.^37^ Kovalchuk *et al*. designed an investigation where human WJ-MSCs were applied in the setting of knee OA in rat models. Polymerase chain reaction (PCR) results showed that the viability of these stem cells is short-lived, but their use in combination with a collagen scaffold increases their residence time.^40^ In summary, multiple preclinical studies show that stem cells derived from WJ could act as a regenerative therapy, but some conflicting evidence between trials still exists. Hence, more investigations should determine whether this form of management is not harmful to humans.

Sadlik *et al*. aimed to determine whether near-perfect cartilaginous tissue could be constructed with the assistance of WJ-MSCs, where the histological, structural and functional properties were preserved, aiming to build a scaffold that integrated in such a way that it was identical to native cartilage. WJ-MSCs are a favourable option, and, when combined with a proper intervention technique and personalized rehabilitation plan, they should be strongly considered for use in immunocompromised patients whose intrinsic regenerative capability is diminished.^41^ Gupta *et al*. described a young patient with grade II knee OA who was injected IA with WJ-MSCs. The individual described attained great symptomatic relief from this intervention, and follow-up studies should be conducted to assess the progression of the condition and pain levels.^42^ Gunay *et al*.’s prospective clinical study determined that patients with knee OA who had been injected with WJ-MSCs reported significant improvements in function and pain. These clinical results were corroborated by MRI and biochemical evidence, by assessing leptin and adiponectin levels.^44^ Samara *et al*. concluded that WJ-MSCs were both safe and effective in patients with knee OA, but the small cohort and lack of long-term data are important limitations to note when interpreting this study.^45^

The clinical studies registered on clinicaltrials.gov aim to establish more definitive evidence supporting the viability of utilizing WJ and/or MSCs derived from it as a promising biologic product for the treatment of knee OA. The aforementioned trials incorporate essential clinical parameters including widely accepted patient reported outcome measures (PROMS), chosen to build the confidence of physicians to utilize this biologic therapy. Researchers should be wary when interpreting the results of these PROMs, as they are subjective parameters, especially pain, which varies between patients. More objective parameters are included in the previously mentioned trials, such as immunomodulatory marker levels and imaging evidence. The ongoing trials aim to recruit a clinically relevant number of patients, ranging from a minimum of 10 to a maximum of 168 patients. Two trials are not yet recruiting, and the status of five trials is currently unknown. All of the studies in question are interventional trials, indicating that they involve the administration of WJ derivatives.

## Conclusion

The present published evidence suggests that WJ tissue and associated MSCs are encouraging for the management of knee OA. The published *in vitro*, preclinical and clinical investigations so far demonstrate the therapeutic potential of WJ and promote further research in this field to provide symptomatic relief to patients suffering from OA, aiming also to regenerate the osteoarthritic hyaline cartilage and exert a disease-modifying effect.

## Data Availability

The data underlying this article are available in the article.
